# Research on design of soft gripper by dielectric elastomer actuator based on quadrangular star honeycomb support

**DOI:** 10.1371/journal.pone.0352654

**Published:** 2026-07-15

**Authors:** Yuanlin Guan, Ziyin Liu, Qi Cheng, Xixin Yang, Chunyu Yu, Yibo Cheng, Xunzhe Gu

**Affiliations:** 1 School of Mechanical and Automotive Engineering, Qingdao University of Technology, Qingdao, Shandong, China; 2 College of Computer Science and Technology, Qingdao University, Qingdao, Shandong, China; Southeast University, CHINA

## Abstract

This study proposes a soft gripper with dielectric elastomer actuator (DEA) and 3D printing quadrilateral star honeycomb support due to the insufficient structural stiffness when grasping irregular objects. According to the bending requirement of the soft gripper and the electrostrictive properties of the dielectric elastomer (DE), a quadrangular star honeycomb structure with zero Poisson’s ratio is proposed as the supporting structure for the soft gripper to enhance its stiffness. The mechanical properties of the honeycomb structure were analyzed via the finite element method (FEM) to improve its stress and horizontal compression displacement. By discussing different parameters such as the pre-stretching ratio of the dielectric elastomer and the pre-compression displacement of the honeycomb structure, we analyzed their influence on the bending displacement performance of the soft gripper. The performance of the soft grippers was verified through both simulations and experiments. The improvement soft gripper, composed of two-layer of dielectric elastomer with a 300% pre-stretching ratio and a honeycomb structure subjected to 20 mm pre-compression, exhibits a bending displacement of 13 mm on a voltage of 5000 V and can grasp objects weighting 5 g.

## 1. Introduction

With the rapid advancement of science and technology, robotic technology has gradually emerged as a pivotal driver of industrial progress, profoundly reshaping industrial production processes and the lifestyles. As a crucial end-effector in robot technology, mechanical grippers were initially applied in fields such as heavy industry production, undertaking simple handling and gripping tasks. In the early stage, mechanical grippers were mostly rigid structures [1], manufactured by rigid materials. They featured high output power, substantial weight, and precise control, yet exhibited poor adaptability when grasping irregular objects. With the development of artificial intelligence and the progress of material science, the rigid gripper gradually develops in the direction of high flexibility and strong flexibility. Flexible grippers are mostly composed of flexible and elastic materials, which have good environmental adaptability and high safety [2]. They mainly solve the problems such as the grabbing of irregular or softer objects [3], which are difficult to be solved by rigid grippers. Therefore, they are widely applied in medical equipment [4], food processing [5], soft robots [6], and agricultural picking [7].

Soft grippers, as a category of flexible gripper, are primarily designed by mimicking the behaviors and motion characteristics of biological organisms. By integrating various actuators, configurations of soft grippers have been developed, including those driven by pneumatic components [8], magnetorheological elastomers [9], shape memory alloys [10], dielectric elastomers [11], and others. Wu et al [12] developed a novel pneumatic soft gripper with an intra-skeletal structure, where inflating a soft rubber component embedded within the fibers caused the gripper to bend, and the intra-skeletal structure to support and transmit the gripping force. Lei et al [13] designed a multi-chamber pneumatic soft gripper featuring an electrical control module, pneumatic control module, sensor module and a human-machine interface. Bernat et al [14] investigated a soft gripper that the iron powder content in the magnetorheological elastomer was changed, and the magnetic properties of the elastomer with an electromagnet switch were adjusted to grasp objects. Kang et al [15] developed an elephant trunk motion-based soft gripper with a flexible polymer exterior and shape memory alloy springs driven internally by the input current, enabling continuous deformation. However, pneumatic grippers suffer from excessive driving force, but the structure is complex. Magnetic field-driven grippers face cost issues, and shape memory alloys exhibit the problems such as slow response and low energy conversion efficiency. Dielectric elastomers offering low cost, fast response and simple actuation, have become a research focus. Due to in-plane electrostriction properties of dielectric elastomer, flexible structural support is required for significant bending deformation of soft gripper. Li et al [16] developed a soft gripper driven by a conical dielectric elastomer, which transmitted displacement and force through a force transmission mechanism, thereby enabling the soft gripper to perform the grasping action. Meng et al [17] developed an elliptical dielectric elastomer soft gripper, which increased the bending angle and output force by adjusting the pre-stretching ratio and the shape. Ci et al [18] researched a soft gripper with a U-shaped support structure made of polyethylene terephthalate **(**PET), and analyzed the multi-branch and multi-segment dielectric elastomer actuator structure of the soft gripper with independent control. Ouyang et al [19] proposed an optimization method for rigid-flexible structure soft grippers with the spring-supported structure. According to the aforementioned researches, the lightweight support structure is difficult to generate a large grasping force, and the hard support structure restricts the deformation of the soft gripper. Therefore, the flexible support structure plays a significant impact for the soft gripper with dielectric elastomers, a nd the design of flexible support structure has an important role on grasping irregular objects with soft gripper such as the dielectric elastomer actuator.

The honeycomb support as a key component of flexible deformed wings for the unmanned aerial vehicle, has the features of the low in-plane stiffness and high out-of-plane (thickness-direction) stiffness, which reduce in-plane stiffness and driving force. When subjected to in-plane compressive loads, it can store energy through compressive deformation. Hence, it has the characteristics such as high out-of-plane rigidity, high loads on the upper and lower surfaces, excellent out-of-plane bearing capacity, and light weight [20]. The elastic modulus and mechanical properties of the honeycomb structure are easily adjusted with the size and shape of the structure [21, 22]. Therefore, it satisfies the widespread applications of various honeycomb structures. Huang et al [23] developed a cosine-type honeycomb structure that demonstrated excellent deformability and enhanced the vehicle’s adaptive ability to fly in multiple conditions. Lin et al [24] designed a concave hexagonal honeycomb structure, and the impacts of some parameters such as wall thickness on load absorption capacity, gradient layer number and gradient distribution on peak stress and specific energy absorption were researched. Ji et al [25] studied a quadrangular star-shaped tubular lattice structure, and analyzed the mechanical properties of the quadrangular star-shaped tubular lattice structure by changing its angle and wall thickness. It showed two deformation modes in different directions under loads. In many honeycomb structures, the quadrangular star honeycomb structure is characterized by fewer structural parameters, symmetry about the origin, simple structure and easy to be improved, so it is suitable for the support of the soft gripper. In addition, Poisson’s ratio is also the key to research the mechanical properties of honeycomb structures. Poisson’s ratios are divided into positive Poisson’s ratio [26], negative Poisson’s ratio [27] and zero Poisson’s ratio [28]. The honeycomb structure with a positive Poisson’s ratio presents a saddle shape and it expands laterally when it is subjected to longitudinal compression. The honeycomb structure with a negative Poisson’s ratio presents a hyperbolic property and it contracts with longitudinal compression [29], while the honeycomb structure with zero Poisson’s ratio does not expand or contract, and the equivalent elastic modulus in the lateral direction will not be affected due to the longitudinal direction. Traditional soft grippers with dielectric elastomers suffer from a limitation in their supporting structure: rigid frames offer good stability but poor compliance, whereas purely soft structures lack sufficient stiffness, making it difficult to achieve both “softness” and “strength” simultaneously. In contrast, honeycomb structures offer significant advantages: they greatly reduce density while maintaining high stiffness and strength. The high porosity enables lightweight design without compromising functionality. The geometric parameters are highly tunable, allowing flexible design. They exhibit excellent energy absorption and resilience, providing reliable support, and their zero Poisson’s ratio endows them with superior compliance. Hence, according to the easy design and zero Poisson’s ratio of the quadrangular star honeycomb structure, it is necessary for soft grippers to design a honeycomb support with in-plane tension and compression stiffness (which can provide a certain support for the pre-stretching ratio of the dielectric elastomer) and an out-of-plane bending stiffness (which can have a good bending performance for the soft gripper).

Therefore, this paper presents a soft gripper integrated with dielectric elastomer actuator and quadrangular star honeycomb support. The primary contributions of this study are as follows:

1) We design a zero-Poisson’s-ratio quadrilateral star-shaped honeycomb structure as the support. The mechanical properties of the honeycomb structure are analyzed, and the honeycomb structure is compressed to store mechanical energy, providing partial force for the bending motion of the soft gripper.
2) The model of the soft gripper is proposed. By systematically analyzing the key parameters including the pre-stretching ratio of the dielectric elastomer and the pre-compression amount of the honeycomb support structure, we improve the overall structural parameters of the soft gripper, significantly enhancing its bending performance.
3) The experiments are conducted to validate the functional effectiveness of the soft gripper, and various irregular objects are successfully grasped.

## 2. Theoretical analysis of dielectric elastomer and single-cell honeycomb structure

### 2.1. Analysis of dielectric elastomer

**Fig 1:**
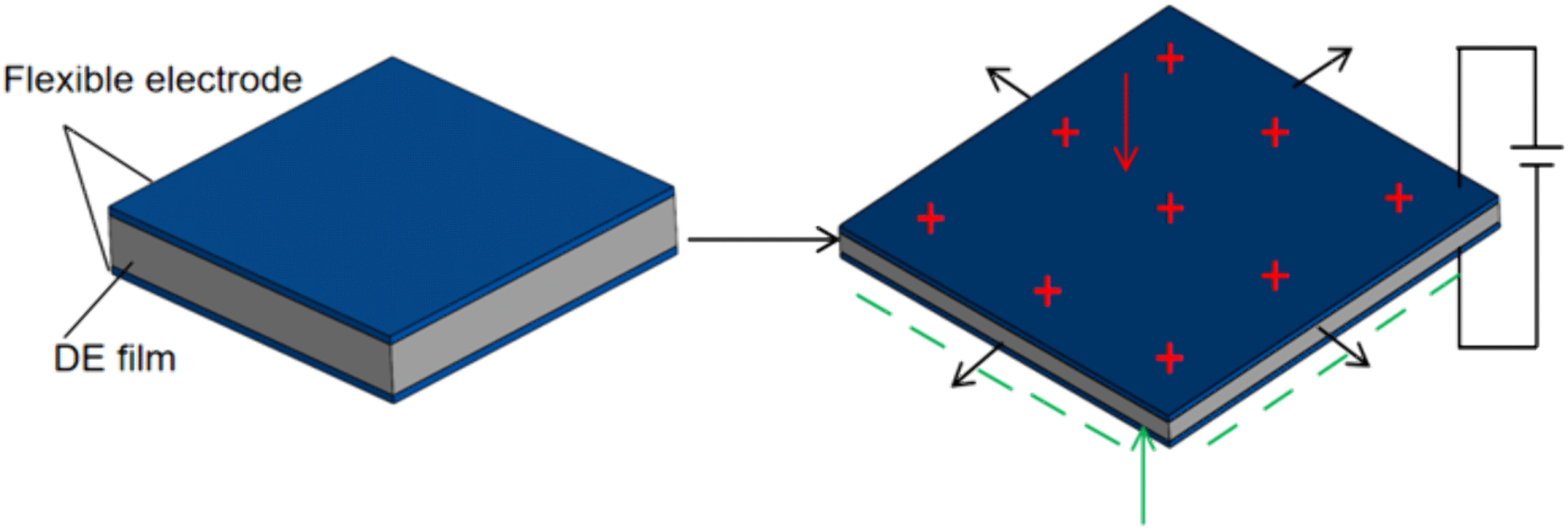
Working principle of dielectric elastomer.

Dielectric elastomers are flexible, intelligent materials that changes the shapes and sizes in response to an electric field, due to their electroactive properties. Notably, they can revert to their original state even with substantial deformation. They have possessing characteristics such as large deformability, low density, and rapid response speed, which are typically coated by flexible electrodes on the upper and lower surfaces. When an electric field is applied, the film undergoes in-plane expansion deformation. The working principle of the dielectric elastomer is illustrated in Fig 1.

Dielectric elastomers exhibit the hyperelasticity, where stress-strain relationships become nonlinear on the applied voltage. Commonly the hyperelastic models include the Neo-Hookean, Gent, Yeoh, and Odgen models [30]. The Neo-Hookean model requires fewer parameters and is suitable for deformation ranges between 30% to 40%, demonstrating the accuracy within limited deformation ranges. As the deformation increases, the predictive power diminishes. The Gent model effectively describes strain hardening during large deformations, applicable across 30% to 150% strain ranges. The Yeoh model provides precise stress-strain characterization for dielectric elastomers beyond 200% strain, but it demands extensive experimental data collection due to its numerous parameters. The Odgen model accurately captures stress-strain relationships in highly elastic materials, operating within 100% to 400% strain ranges with minimal parameters. The model not only facilitates experimental measurements but also effectively addresses strain hardening phenomena during significant deformation.

Given that dielectric elastomers exhibit pronounced large-strain characteristics on the electroactive performance and can undergo substantial area in the under equibiaxial deformation, this study employs the Ogden model to simulate their deformation behavior. Compared with other constitutive models, the Ogden model offers higher fitting accuracy in large-strain simulations and demonstrates superior predictive capability under equibiaxial loads, thereby enabling accurate characterization of the deformation response of dielectric elastomers. Through mechanical experiments, stress–strain data of the dielectric elastomer under large deformations are obtained [19]. The analytical expression of the Ogden constitutive model is established, and the model parameters are determined by curve fitting using the least-squares method in conjunction with experimental data from specific loading conditions, thereby characterizing the material’s nonlinear hyperelastic behavior under large deformations.

The function expression of Odgen model [31]:


$$\begin{array}{c} \begin{array}{c} W=\sum_{i=\text{1}}^{N}\frac{\mu_{i}}{\alpha_{i}}\left(\overset{\alpha_{i}}{\underset{\text{1}}{\lambda }}+\overset{\alpha_{i}}{\underset{\text{2}}{\lambda }}+\overset{\alpha_{i}}{\underset{\text{3}}{\lambda }}-\text{3}\right) \end{array} \end{array}$$ (1)

Among them, *λ*_*1*_, *λ*_*2*,_ and *λ*_*3*_ are the principal stretch ratio of the material, and *μ*_*i*_ and α_*i*_ are material parameters, *W* is the strain energy density function, and *N* is the order of the model.

### 2.2. Mechanical theoretical analysis of single-cell quadrilateral star honeycomb structure

#### 2.2.1. Theoretical analysis of the transverse equivalent elastic modulus of honeycomb structure.

**Fig 2:**
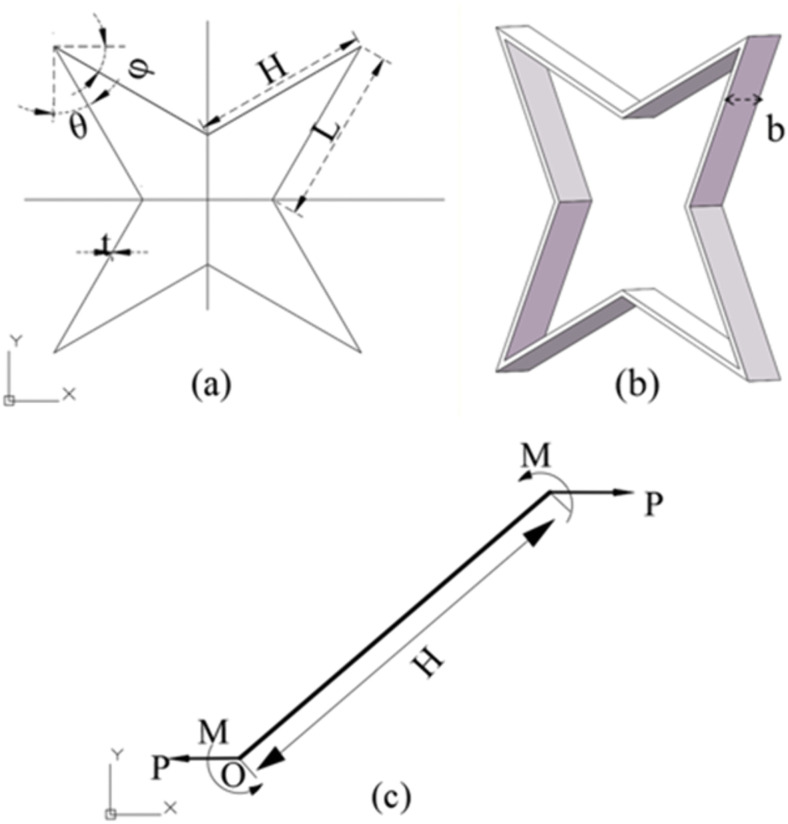
Quadrilateral star-shaped zero Poisson’s ratio honeycomb structure diagram. (a) Plan view of quadrilateral star-shaped honeycomb structure, (b) three-dimensional view of honeycomb structure and (c) force analysis diagram.

The honeycomb structure exhibits symmetry and periodicity. Based on homogenization theory, this study analyzes the transverse equivalent elastic modulus of the honeycomb structure by focusing on its unit cell—quadrangular star honeycomb structure with a zero Poisson’s ratio, as illustrated in Fig 2. The honeycomb structure is a center symmetric structure. The lengths and the thickness of honeycomb wall and the overall thickness of honeycomb are represented by *H*, *L*, *t*, *θ*, *φ* and *b*, respectively (as shown in Fig 2 (a) and (b). The elastic modulus of honeycomb material is *E*_*s*_, and the dimensionless parameters *α*, *β* and *γ* are defined, where *α* = *H/L*, *β* = *t/L*, *γ* = *b/L*. A honeycomb wall is selected for stress analysis according to the symmetric structure of the honeycomb cell, as shown in Fig 2 (c). The honeycomb wall is affected by an external force *p* and a pair of bending moments *M* in the x direction. o is the origin, *s* is the distance from the origin, and the honeycomb wall has lateral and bending deformation.

Based on the moment balance condition at the origin on the left end, the bending moment *M*_*s*_ on any section at a distance from the origin can be obtained as follows:


$$\begin{array}{c} M_{s}=s\cdot P\sin\varphi -M \end{array}$$ (2)

where *M* is the bending moment at the origin.

The external force *P* in the horizontal direction is:


$$\begin{array}{c} \begin{array}{c} P=\sigma bL\text{cos}\theta \end{array} \end{array}$$ (3)

Where *σ* is the stress in the x-direction.

The angle of the cross-section at any distance from the origin is:


$$\begin{array}{c} \begin{array}{c} \Delta \left(\text{s}\right)=\int \frac{M_{s}}{E_{s}I}+c \end{array} \end{array}$$ (4)

According to the symmetry of the structure, the boundary conditions for the corners at both ends of the honeycomb inclined wall are:


$$\begin{array}{c} \begin{array}{c} \Delta \left(\text{0}\right)=\text{0},\Delta \left(H\right)=\text{0} \end{array} \end{array}$$ (5)

The boundary are substitute into the corner formula of the cross-section at any distance from the origin, and the *M* is:


$$\begin{array}{c} M=\frac{HP\sin\varphi }{2} \end{array}$$ (6)

Applying the principle of virtual work, a unit virtual load is applied in the x direction, causing the honeycomb cell walls to undergo both bending and axial deformation. The displacement of the honeycomb wall in the x direction is:


$$\begin{array}{c} \begin{array}{c} \delta =\left(\int \frac{M_{s}M_{\text{1}}}{E_{s}I}\text{d}s\right)\text{sin}\varphi +\left(\int \frac{F_{s}F_{\text{1}}}{E_{s}A}\text{d}s\right)\text{cos}\varphi \end{array} \end{array}$$ (7)

where *δ* is the displacement of the honeycomb wall in the x-direction, *M*_*s*_ and *F*_*s*_ represent the actual loads acting on the honeycomb wall, while *M*_*1*_ and *F*_*1*_ represent the unit virtual loads acting on the honeycomb wall. The expressions for each load are:


$$\begin{array}{c} M_{s}=s\cdot P\sin\varphi -\frac{HP\;\sin\varphi }{2} \end{array}$$ (8)

$$\begin{array}{c} M_{I}=s\cdot \sin\varphi -\frac{H\sin\dot{\varphi }}{2} \end{array}$$ (9)

$$\begin{array}{c} \begin{array}{c} F_{s}=P\text{cos}\varphi \end{array} \end{array}$$ (10)

$$\begin{array}{c} \begin{array}{c} F_{\text{1}}=\text{cos}\varphi \end{array} \end{array}$$ (11)

By substituting the expressions for each load, the end displacement of the honeycomb wall can be obtained as:


$$\begin{array}{c} \begin{array}{c} \delta =\frac{P\alpha^{\text{3}}L^{\text{3}}{\text{sin}}^{\text{3}}\varphi }{\text{12}E_{s}I}+\frac{P\alpha L{\text{cos}}^{\text{3}}\varphi }{E_{s}A} \end{array} \end{array}$$ (12)

where *I* is the moment of inertia and *A* is the cross-sectional area of the honeycomb wall;

Therefore, the strain *ε* of the honeycomb wall in the x direction is:


$$\begin{array}{c} \begin{array}{c} \epsilon =\frac{\delta }{H\text{cos}\varphi } \end{array} \end{array}$$ (13)

The equivalent elastic modulus *E*_*x*_ of the honeycomb wall in the x direction is:


$$\begin{array}{c} \begin{array}{c} E_{x}=\frac{\sigma }{\epsilon } \end{array} \end{array}$$ (14)

By substituting the stress *σ* and strain *ε* into the formula (14), the dimensionless equivalent elastic modulus can be obtained as:


$$\begin{array}{c} \frac{E_{x}}{E_{s}}=\frac{\beta^{3}\cos\varphi }{\cos\theta \left(\alpha^{2}\sin^{3}\varphi +\beta^{2}\cos^{3}\varphi \right)} \end{array}$$ (15)

#### 2.2.2. Theoretical analysis of poisson’s ratio for quadrilateral star honeycomb support.

The quadrangular star honeycomb structure is central symmetric. The corner angles of the honeycomb structure tend to zero on the unidirectional load. Specifically, under x-direction stress, the y-direction strains at the honeycomb’s top regions cancel each other out—rendering the x-direction and y-direction deformations of the structure mutually independent. Since y-direction strain is zero when the honeycomb is loaded in the x direction, the Poisson’s ratio of the quadrangular star honeycomb structure is determined to be zero by the formula (16). Therefore, it is confirmed as a zero Poisson’s ratio structure.


$$\begin{array}{c} \begin{array}{c} \upsilon =-\frac{\epsilon^{\prime }}{\epsilon } \end{array} \end{array}$$ (16)

where *υ* is Poisson’s ratio and *ε’* is the strain in the y-direction.

## 3. Structural analysis of soft gripper

To achieve effective synergy between the dielectric elastomer and the honeycomb structure, we first conducted the finite element analysis on the dielectric elastomer film to determine its pre-stretching ratio. Subsequently, we perform the models and multiple rounds of structural improvement on the honeycomb structure, ultimately obtaining the configuration with the best performance. Hence, we carry out pre-compression simulations of the honeycomb structure, and in conjunction with the stress characteristics of the dielectric elastomer film on the pre-stretching conditions, comprehensively evaluate and determine the most suitable pre-compression range for the honeycomb structure.

### 3.1. Mechanical simulation analysis of dielectric elastomer

The pre-stretching configuration not only enables the film to exhibit excellent deformability but also provides the pre-stretching force for the mechanical simulation of the soft gripper. Therefore, the equibiaxial pre-stretching deformation of the dielectric elastomer is simulated. This study adopts the VHB4910 film manufactured by 3M Company, with a thickness of 1 mm and an initial dimension of 80 mm × 80 mm × 1 mm.

**Fig 3:**
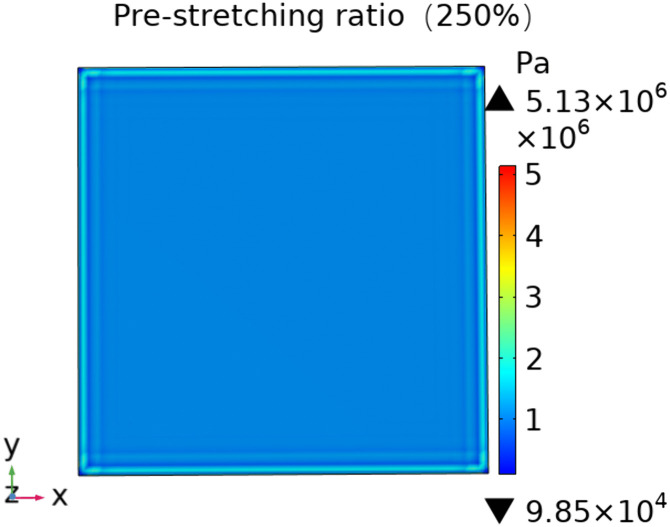
Simulation diagram of the biaxial stress with 250% pre-stretching ratio for dielectric elastomer.

**Fig 4:**
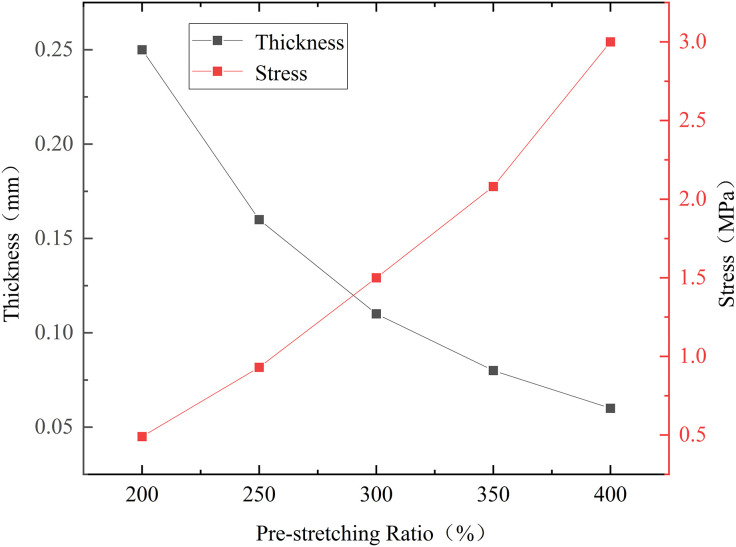
Simulation results of thickness and stress with different pre-stretching ratios.

A geometric simulation model of the dielectric elastomer is constructed. Following an analysis of hyperelastic models, the Ogden model is selected, and the parameters are presented in Table 1. In accordance with the actual equibiaxial stretching process, equal specified displacement constraints are applied to its four boundaries. The pre-stretching effects of the film are simulated, with pre-stretching ratios set at 200% to 400% in 50% increments. Fig 3 presents the biaxial stress simulation results at a 250% pre-stretching ratio, while Fig 4 illustrates the relationships between thickness/stress and the pre-stretching ratio.

**Table 1:** Parameters of the Odgen model [19].

Property	Value	Unit
**Shear modulus *μ***_**1**_	24940	Pa
**Shear modulus *μ***_**2**_	57340	Pa
**Shear modulus *μ***_**3**_	−57400	Pa
**Parameter *α***_**1**_	1.2814	–
**Parameter *α***_**2**_	3.0164	–
**Parameter *α***_**3**_	3.0158	–

As illustrated in Fig 3, the maximum stress of the film is 5.13 × 10⁶ Pa, and the average stress is calculated to be 9.4 × 10⁵ Pa. While slight stress variations exist across the film, the stress distribution in the middle region is uniform. Thus, the middle part of the film is selected as the target area for the biaxial pre-stretching experiment. In Fig 4, it can be observed that as the pre-stretching ratio increases, the film thickness decreases gradually while the stress increases. Notably, beyond a certain threshold, the film stress rises sharply—it increases the risk of the breakdown when a voltage is applied. Based on the above findings, the pre-stretching ratio of the film is set within the range of 250%–300% for subsequent experiments.

### 3.2. Simulation of zero poisson’s ratio for quadrangular star honeycomb structure

**Fig 5:**
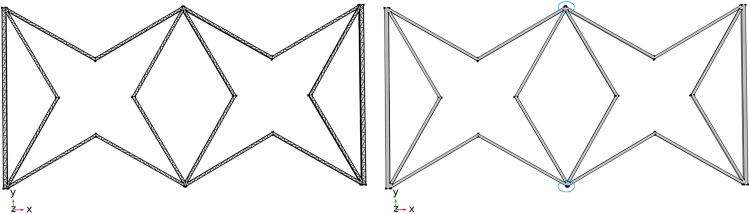
Mesh and probe application diagram for the quadrangular star-shaped honeycomb structure.

A simulation model of a quadrangular star-shaped honeycomb structure is constructed, and the mesh is shown in Fig 5. The model is a 1 × 2 single cell structure. The parameters of the single honeycomb structure are as follows: honeycomb wall length *H* = *L* = 25 mm, honeycomb angle *φ* = *θ* = 30°, and honeycomb wall thickness *t* = 0.6 mm.

With the widespread adoption of 3D printing technology, polylactic acid (PLA) and thermoplastic polyurethane (TPU) have become commonly used materials. However, in the design of support structures for dielectric elastomer actuators (DEAs), stiffness and dimensional stability are critical. The pre-stretching performance of the DEAs is required to enhance electroactive deformation, which generates a continuous contractile force. The support structure must therefore possess high rigidity to resist deformation and prevent creep.

PLA offers excellent creep resistance, high modulus, and good dimensional stability, effectively locking in the pre-stress and ensuring long-term actuation reliability. In contrast, although TPU can be printed into complex geometries such as honeycomb structures, its low stiffness and high elasticity cause the structure to become overly soft. Hence it is unable to provide adequate support. Additionally, TPU is more challenging to print and difficult to post-process, further limiting its suitability for load-bearing frames. Therefore, for the honeycomb support structure in soft grippers, PLA is preferred to balance rigidity, printing precision, and actuation performance—enabling both flexible bending and reliable gripping force.

**Fig 6:**
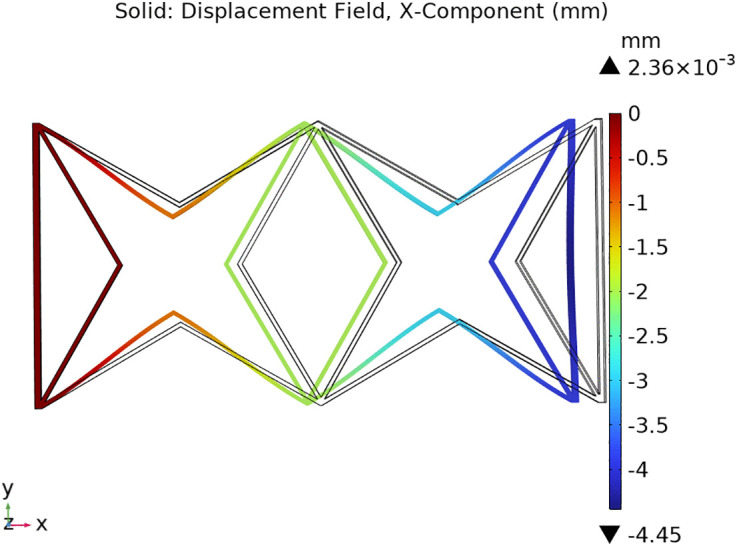
Compression simulation of honeycomb structure in the x direction.

PLA is selected as the material for the honeycomb structure. The boundary conditions are defined as follows: the left end of the model is fixed, while the upper and lower ends are set as free boundaries. When a horizontal force (applied from right to left) is exerted on the structure, the honeycomb undergoes leftward horizontal displacement. Based on stress distribution of the film (Fig 4) and an analysis of the honeycomb’s horizontal cross-sectional area, the applied horizontal force is set as 1 N. Therefore, the horizontal (x-direction) displacement of the honeycomb structure is obtained as 4.45 mm on the numerical calculation, as illustrated in Fig 6. To measure the vertical displacement at the upper and lower boundary vertices, point probes are placed at the upper and lower intersection points of two adjacent honeycomb unit cells (blue circle part in Fig 5), yielding a vertical (y-direction) displacement of 4.6 × 10^-^³ mm.

Based on the horizontal and vertical strains of the pre-compression honeycomb structure, the Poisson’s ratio of the honeycomb structure is calculated. According to the horizontal and vertical simulation displacements, the strains in the x and y-directions are 5.6 × 10^−2^ and 1.2 × 10^−4^, respectively. Based on the above strains, the Poisson’s ratio in formula (16) is 2.1 × 10^−3^, which is almost zero. Therefore, the quadrangular star-shaped honeycomb structure has zero Poisson’s ratio.

### 3.3. Simulation of improved honeycomb structure

**Fig 7:**
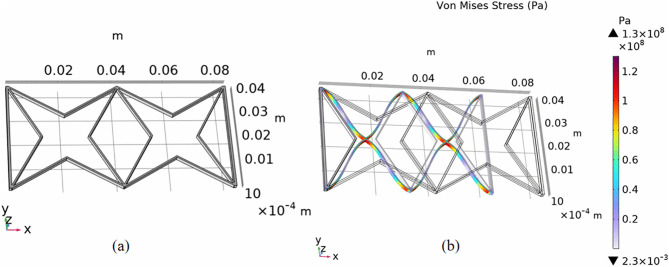
Simulation diagram of first honeycomb structure(a) the origin diagram, and b) the diagram under the load.

To enable the honeycomb structure to support the pre-stretching force of the film while minimizing secondary deformation, a pre-compression simulation analysis of the honeycomb structure is conducted. The structure is then improved to achieve a configuration compatible with the film. As illustrated in Fig 7, the left end of the honeycomb structure is fixed, while a x-direction displacement constraint is applied to the right end. The structural parameters are as follows: the arc radius at the vertex of each honeycomb cell is 0.4 mm. Both the length and width of a single cell are 42 mm. The total x-direction length of the honeycomb structure is 84 mm, and the honeycomb angles satisfy *φ* = *θ* = 30°.

From Fig 7, it can be seen that there is the maximum stress in the red part of honeycomb structure, and the maximum stress is 1.3 × 10^8^ Pa. According to the derived value, the average stress of the whole honeycomb structure is 1.1 × 10^7^ Pa, most of which occurs at the vertex or corner, resulting in stress concentration, which may lead to fatigue failure or fracture of the structure. For the first honeycomb structure, so that the honeycomb structure can support the pre-stretching ratio of the film, theoretically, and the stress of the honeycomb structure shall be kept in the same order of magnitude as the stress of the film. However, the stress of the honeycomb structure is too large. The rigidity of the honeycomb structure on a certain amount of honeycomb compression is too large, and the equivalent elastic modulus of the first honeycomb structure is 4.5 × 10^7^ Pa.

**Fig 8:**
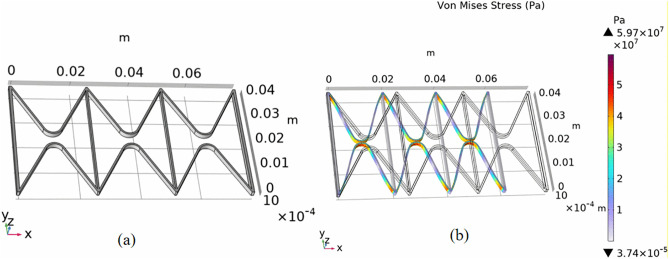
Simulation diagram of second honeycomb structure(a) the origin diagram, and b) the diagram under the load.

In order to solve the problems such as the stress concentration, large stress and stiffness of the above honeycomb structure, the structure is improved. The cell length of the new structure is 25 mm and the width is 42 mm. To ensure the compressibility of the honeycomb structure, the number of cells of the honeycomb structure is increased to 3, and the total length of both ends of the honeycomb structure in the x direction is 78 mm. Based on the analysis of the above structure, the four walls around the honeycomb cell are changed into two vertical walls, and *φ* is changed from 30° to 60°. For the large intermediate stress of the first structure, the intersection of the two honeycomb walls in the middle is changed into an arc transition, and the arc radius is 3 mm. The stress simulation diagram of the second honeycomb structure is obtained, as shown in Fig 8.

From Fig 8, the maximum stress is 5.97 × 10^7^ Pa, and the stress concentration phenomenon has been greatly weakened. According to the derived value, the average stress of the whole honeycomb structure is 7 × 10^6^ Pa, and the equivalent elastic modulus of the second honeycomb structure is 2.7 × 10^7^ Pa. Compared with the first result, the rigidity of the honeycomb structure is reduced. However, it is found that the distance between the two vertices at the upper and lower edges is large, and the hollow part in the middle of the honeycomb may not support the shape of the film, causing the film at the hollow part to shrink inward. In addition, the two peaks at the upper and lower edges of the honeycomb structure are relatively sharp, causing the film to break.

**Fig 9:**
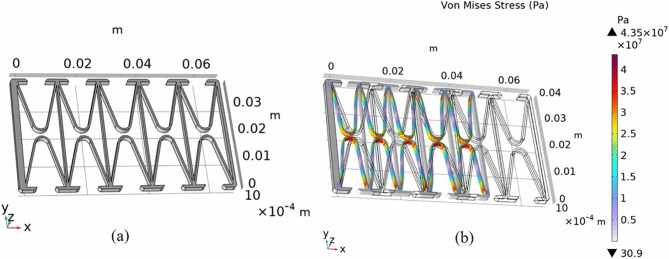
Simulation diagram of the third honeycomb structure(a) the origin diagram, and b) the diagram under the load.

To solve the above problem, the second honeycomb structure is improved, and a third honeycomb structure is designed. The cell length of the structure is 13 mm and the width is 40 mm. The number of cells for the honeycomb structure is increased to 5, and the total length of the two ends of the honeycomb structure in the x-direction is 70 mm. Based on the improvement of the second structure, the *φ* angle is increased to 75°, and a short plate is added at the vertex of the upper and lower edges of the honeycomb structure in order to fix the fixture and compress. The length of the short plate is 6 mm, and the right angle of the short plate is treated with an arc. Fig 9 shows the simulation diagram of the third quadrilateral multicell honeycomb structure.

From Fig 9, the maximum stress is 4.35 × 10^7^ Pa, and the average stress of the whole honeycomb structure is calculated as 4.5 × 10^6^ Pa through the derived value. The equivalent elastic modulus of the third honeycomb structure is 1.6 × 10^7^ Pa. The stress and rigidity of the third honeycomb structure are reduced, and the average stress of the pre-compression honeycomb structure and is in the same order of magnitude with the stress of pre-stretching film, so the honeycomb structure meets the support requirements of the film. With the improved honeycomb structure compressed, the distance between every two points at the edges of the honeycomb structure becomes smaller, and the degree of film contraction inward decreases. With the addition of boards at the upper and lower edges, the film will not break due to the sharp points, and the boards provide support to prevent the film from shrinking in the vertical direction (y-direction). Therefore, the third multi-cell honeycomb structure of the quadrangular star is used as the final support structure.

### 3.4. Fatigue strength analysis of 3D-Printed PLA honeycomb structures

#### 3.4.1. Study on fatigue simulation based on S-N curve.

The S-N curve is the core of fatigue analysis. Its principle is that the material fatigue life decreases with the increase of stress amplitude, and an infinite service life can be achieved when the stress is lower than the fatigue limit. Restricted by experimental conditions, experimentally verified S-N curve data is directly adopted as the input for this simulation [32]. Since brittle materials such as PLA exhibit negligible plastic deformation and approximately linear fatigue behavior, the S-N curve criterion is selected in the fatigue module with Goodman mean stress correction for simplification [33]. The ultimate tensile strength is 60 MPa, and the equivalent stress formula is expressed as follows:


$$\begin{array}{c} \begin{array}{c} \sigma_{eq}=\frac{\sigma_{a}}{\text{1}-\frac{\sigma_{m}}{\sigma_{b}}}\#(\text{1}\text{7}) \end{array} \end{array}$$ (17)

where $\sigma_{a}$ is the stress amplitude, $\sigma_{m}$ is the mean stress, and $\sigma_{b}$ is the ultimate tensile strength.

#### 3.4.2. Analysis of fatigue life simulation results.

**Fig 10:**
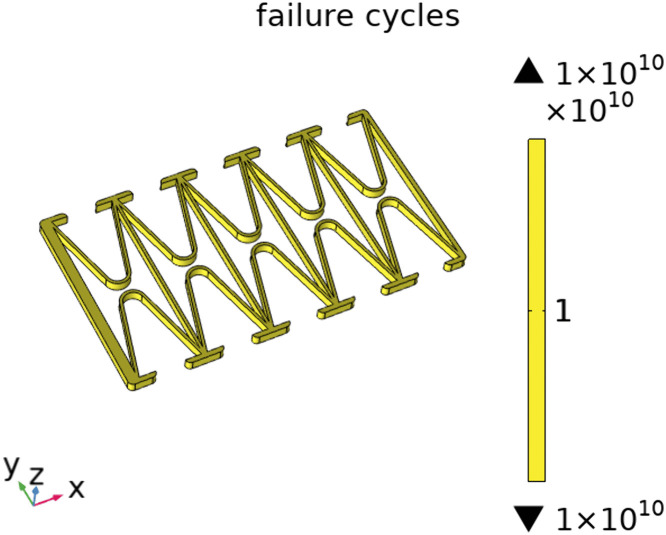
Failure cycles diagram of honeycomb structure under 1 N load.

**Fig 11:**
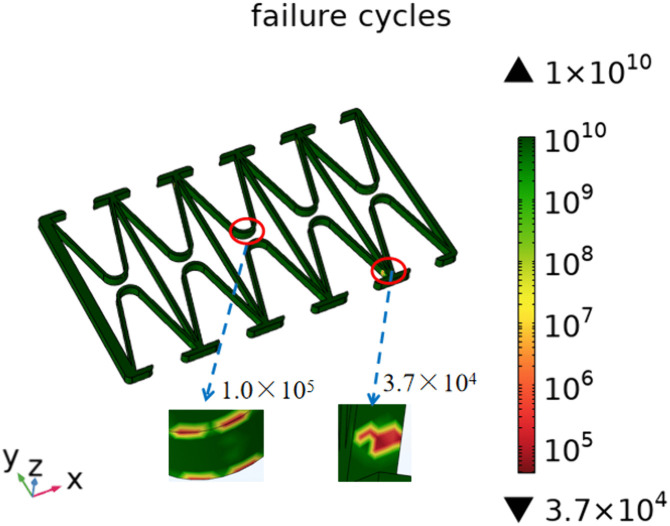
Failure cycles diagram of honeycomb structure under 1.5 N load.

To study the fatigue life of honeycomb structures, the optimized model is imported into COMSOL. One end is fixed, while a cyclic compressive load is applied on the other end. The simulation results are presented in Figs 10 and 11.

Fig 10 shows that the structure exceeds 10^10^ failure cycles under an external load of 1 N, which means that fatigue damage rarely occurs under normal operating loads. As shown in Fig 11, when the load increases to 1.5 N, two fracture-prone regions are observed on the structure. The failure cycles at the edge root and the middle bending section decrease to 3.70 × 10^4^ and 1.0 × 10^5^, respectively.

**Fig 12:**
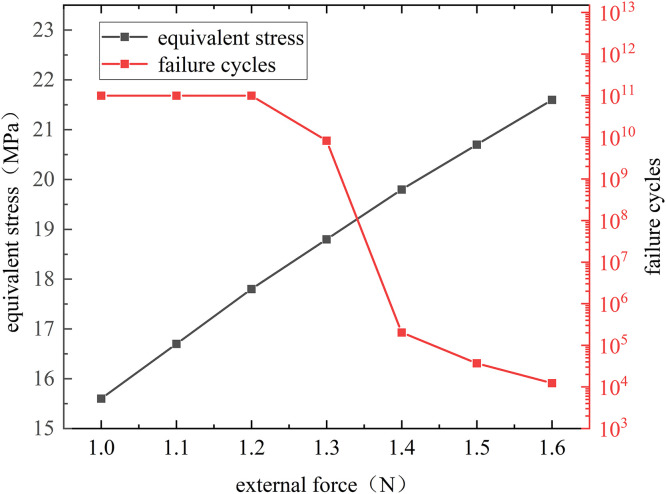
S-N curve of honeycomb structure under different external loads.

For compressive loads ranging from 1 N to 1.6 N, the equivalent stress values and corresponding fatigue failure cycles of the honeycomb structure are systematically collected. The S-N curve of the honeycomb structure is presented in Fig 12.

Under normal operating pulsating load of 1 N external force, the maximum stress is 42.1 MPa and the minimum stress is 0, which are substituted into the following formulas for mean stress $\sigma_{m}$ and stress amplitude $\sigma_{a}$.


$$\begin{array}{c} \begin{array}{c} \sigma_{m}=\frac{\sigma_{max}+\sigma_{min}}{\text{2}}\#(\text{1}\text{8}) \end{array} \end{array}$$ (18)

$$\begin{array}{c} \begin{array}{c} \sigma_{a}=\frac{\sigma_{max}-\sigma_{min}}{\text{2}}\#(\text{1}\text{9}) \end{array} \end{array}$$ (19)

Substitute the calculated values into the Goodman equivalent stress formula, and the obtained equivalent stress $\sigma_{eq}$ is 15.6 MPa. Compared with the S-N curve, its fatigue life exceeds 10^10^ cycles, which verifies the accuracy of the simulation results.

Under the pulsating working load of 1.5 N, the maximum stress is 63.1 MPa and the minimum stress is 0. Following the same calculation process using the mean stress, stress amplitude, and the Goodman equivalent stress expression, the resulting $\sigma_{eq}$ is 20.7 MPa. The S-N curve indicates a fatigue life on the order of 10^4^ cycles, further validating the correctness of the simulation.

When subjected to pulsating working loads above 1.6 N, the component will fail rapidly. Therefore, loads exceeding 1.6 N should be avoided in daily application.

### 3.5. Simulation analysis of bending performance of soft gripper

#### 3.5.1. Simulation model of a single soft gripper.

**Fig 13:**
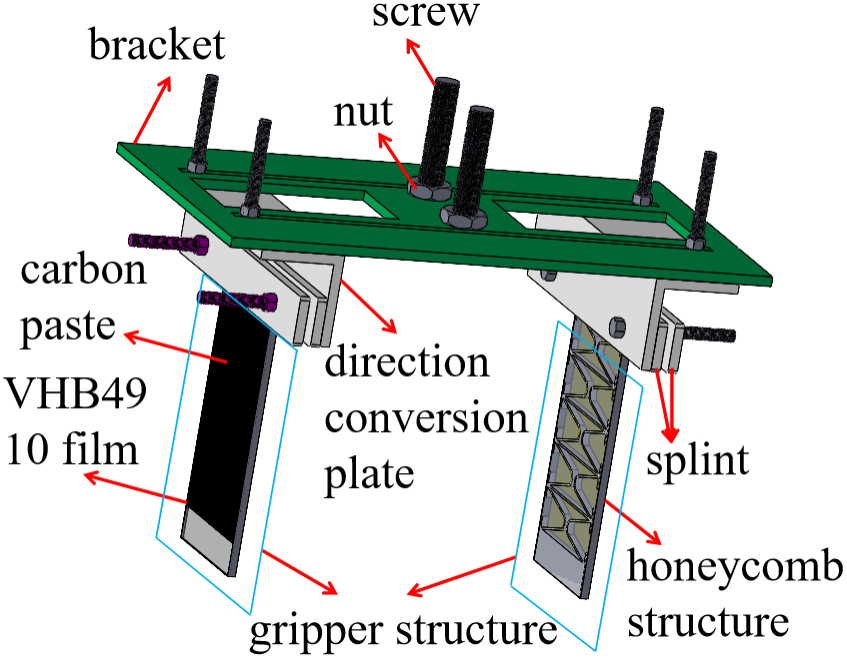
Schematic diagram of the soft gripper.

Based on the above honeycomb support structure, a soft gripper is designed, as shown in Fig 13. The soft gripper is composed of two single grippers with the same structural parameters. A single gripper consists of VHB4910 film, honeycomb structure, carbon paste, and the conductor. Each gripper holds a fixed end of the gripper by a splint, and it is fixed to a direction conversion plate by a screw and a nut. The direction conversion plate is fixed to a bracket, and the direction conversion plate has a left-right movement on the bracket rail, so that the distance between the gripper structures can be adjusted according to the shape of the object.

**Fig 14:**
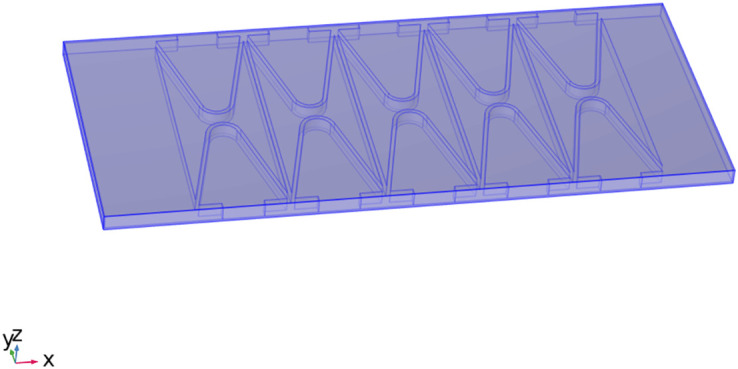
Finite element model of a single gripper.

**Fig 15:**
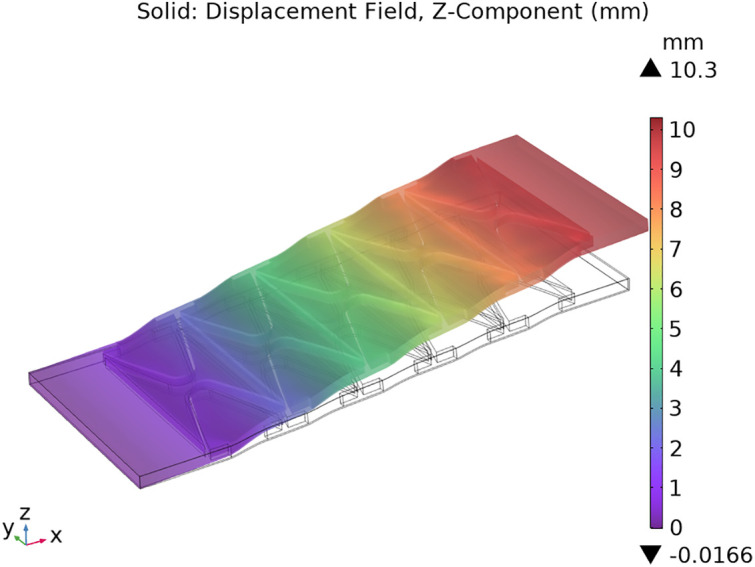
Simulation diagram of a single soft gripper.

To analyze the mechanical properties of the soft gripper, a finite element model of a single gripper is built as shown in Fig 14. The length and width of the individual gripper are set as 90 mm and 40 mm, respectively, and five cells arranged in a honeycomb structure are featured. The long plates are attached to each end of the honeycomb structure: one end is fixed in the clamp and the other serves as the gripping surface. The entire structure is wrapped in a one-layer film. When the voltage is applied to the gripper, the gripper undergoes bending deformation due to three combined forces: pre-stretching force from the film, pre-compression force from the honeycomb structure, and Maxwell’s stress. Based on simulation parameters, the film’s pre-stretching ratio and the honeycomb structure’s pre-compression displacement are set at 300% and 20 mm, respectively. The film-coated electrode has its outer layer as the ground and an internal 3000 V voltage is applied. Boundary conditions specify a fixed left end and a free right end. The simulation results for a single soft gripper are presented in Fig 15.

As illustrated in Fig 15, the designed single soft gripper finger can generate bending deformation, achieving a maximum bending displacement of 10.3 mm. It is found that the pre-compression amount of the honeycomb structure and the pre-stretching ratio of the film exert significant impacts on the bending displacement. The two parameters—the film’s pre-stretching ratio and the honeycomb structure’s pre-compression displacement—can effectively improve the grasping performance of the soft gripper.

#### 3.5.2. Mechanical simulation analysis with the different pre-compression displacement of honeycomb structures.

**Fig 16:**
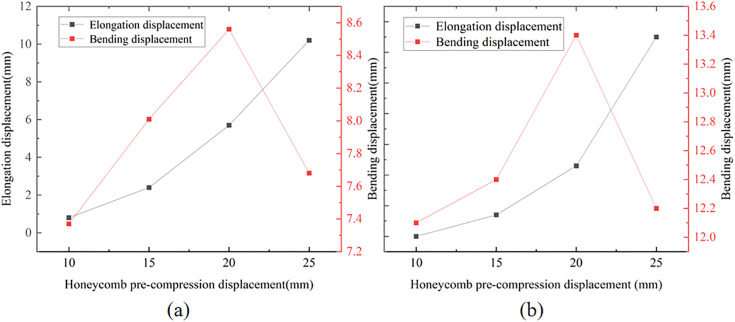
Mechanical simulation of a single soft gripper with different layers for the film on different pre-compression displacements. (a) A one-layer film. (b) a two-layer film.

To obtain the range of the pre-compression displacement of the honeycomb structure, it is known from the previous simulation of the film that the pre-stretching ratio range of the selected film is 250% to 300%. Therefore, the pre-stretching ratio of the fixed film is 300%, and the simulation analysis of a single soft gripper is conducted. The relationships between the pre-compression displacement of the honeycomb structure and the displacements (the elongation and bending displacement) are shown in Fig 16.

Fig 16 shows (a) the simulation results of a one-layer film-coated and (b) a simulation of a two-layer film-coated. The black lines in the figure reveal that the elongation displacement of the single grippers increases with the honeycomb structure’s pre-compression displacement. Because the contraction force of the film remains constant, so the honeycomb’s restoring force increases. Additionally, since the contraction force of the one-layer film is less than that of the two-layer film, the elongation displacement of the one-layer structure is greater than that of the two-layer. The red curves in the figures show that the bending displacement of the soft gripper first increases and then decreases as the honeycomb’s pre-compression displacement increases. The maximum displacement occurs when the pre-compression displacement reaches 20 mm. The displacement of the soft gripper’s elongation also increases with the increase of pre-compression displacement, but because the film’s thickness becomes too thin, it is prone to breakdown and consequently the bending displacement reduces.

#### 3.5.3. Mechanical simulation analysis with different pre-stretching ratios of dielectric elastomer.

**Fig 17:**
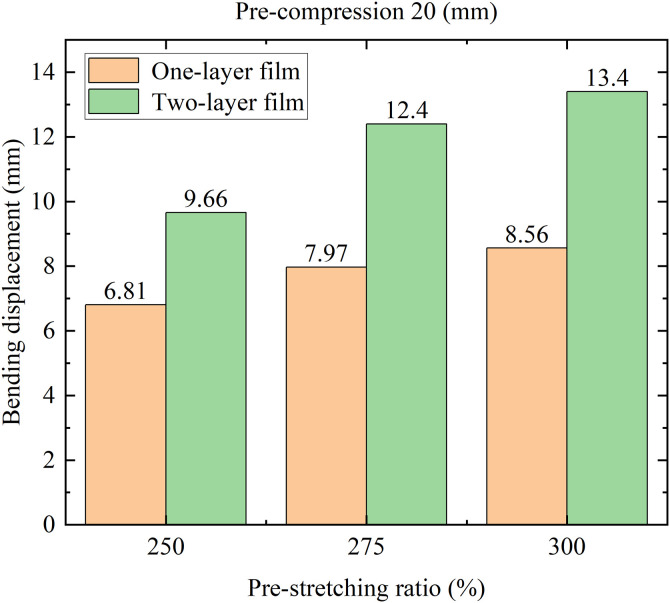
Simulation data chart under different pre-stretching ratios.

As shown in Fig 16, the bending displacement of the soft gripper reaches the maximum when the pre-compression displacement of the honeycomb structure is 20 mm. Simulation analysis is discussed on different film pre-stretching ratio, as shown in Fig 17.

As shown in Fig 17, the bending displacement increases with the pre-stretching ratio of the film, reaching the maximum at 300%. The maximum bending displacement of the soft gripper is 13.4 mm. Soft grippers made from two-layer films exhibit greater bending displacement compared to those constructed with the one-layer film.

## 4. Experimental results and discussion of the soft gripper

Based on the above simulation analysis and structural improvement, the following design constraints are established: the pre-stretching ratio of the dielectric elastomer film is maintained within 250%–300%, and the pre-compression displacement of the honeycomb structure is set as the range of 15 mm and 20 mm. Previous simulations of a single soft finger demonstrated that the proposed design can achieve effective bending deformation. To further enhance the maximum bending displacement of the soft gripper, a parameter-combination improvement approach is proposed: within the aforementioned constraint ranges, dielectric elastomer films with different pre-stretching ratios are paired with honeycomb structures featuring varying pre-compression levels, and a series of systematic bending experiments are conducted. Compared with the measured bending displacements, the parameter combination yielding the best bending performance is identified. Finally, a soft gripper is fabricated by these improved parameters and subjected to grasping validation experiments.

### 4.1. Preparation of soft gripper

**Fig 18:**
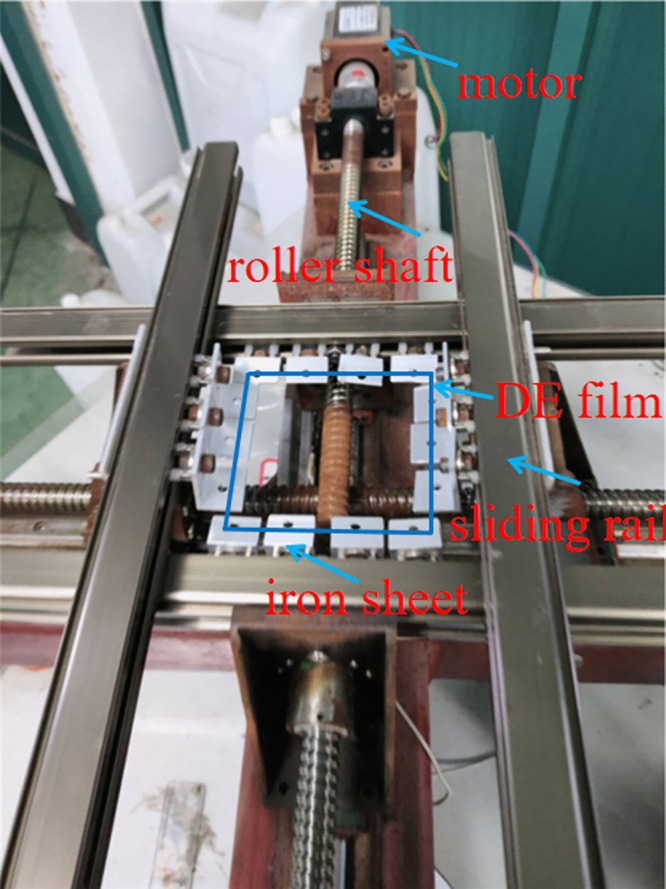
Equibiaxial pre-stretching machine of dielectric elastomer.

The soft gripper is manufactured by 3D printing machine according to the designed honeycomb structure, and PLA material is used. The film is pre-stretched by an equal biaxial pre-stretching machine, as shown in Fig 18. A square-cut film placed onto the iron sheets arranged around the perimeter. The motor drives two roller shafts to rotate, and the sliding rails move outward simultaneously, causing the iron sheets to slide synchronously along the rails. Thereby the equibiaxial pre-stretching force is applied to the film.

**Fig 19:**
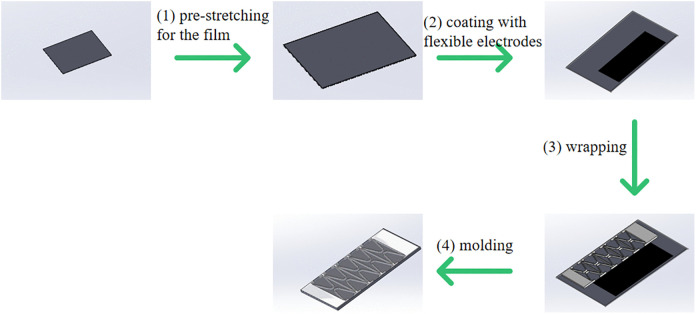
Design and preparation process of a single soft gripper.

The preparation of a single soft gripper can be divided into four steps: (1) pre-stretching for the film; (2) coating with flexible electrodes; (3) wrapping; and (4) molding. The design and preparation process of a single soft gripper is shown in Fig 19.

### 4.2. Experimental platform construction for mechanical property test of soft gripper

**Fig 20:**
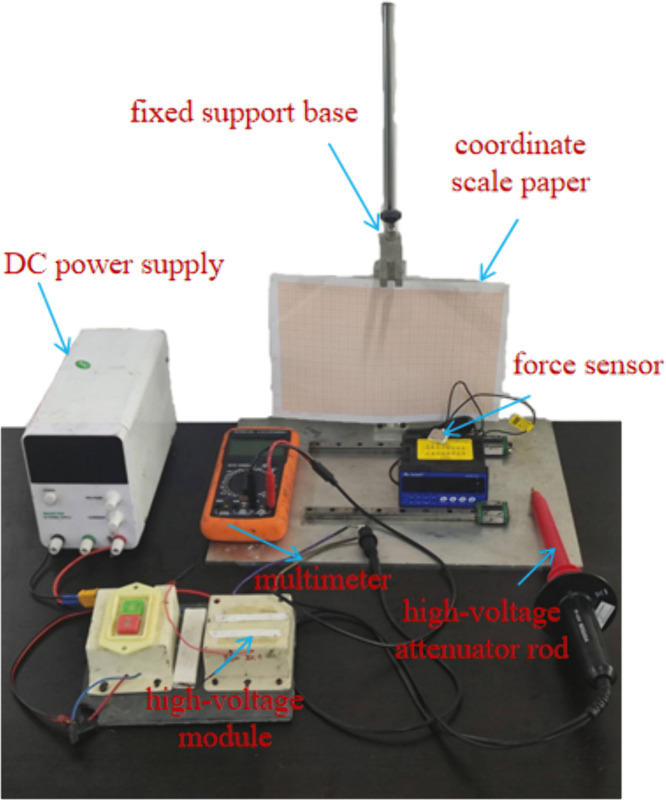
Electro-induced experimental platform.

To test whether a soft gripper can achieve bending deformation for grasping objects, we construct a testing platform of the mechanical performance as shown in Fig 20. The platform consists of the following components: direct current (DC) power supply, high-voltage module, multimeter, high-voltage attenuator rod, fixed support base, coordinate scale paper, and force sensor. The DC voltage is amplified through the high-voltage module and applied to the film; causing deformation. Because the bending displacement of the soft gripper is large, so the coordinate scale paper is applied to measure the value. The high-voltage attenuator rod reduces the high voltage to a lower level, and the applied voltage value is read out through a multimeter.

The high voltage required for electro-deformation of the film is amplified by the high voltage module by 2000 times, and the multimeter is used to measure the actual voltage of the soft gripper. However, the multimeter cannot measure the voltage above 1000 V, so the high-voltage attenuation rod is required to attenuate the actual voltage for measurement. The high-voltage attenuation rod can reduce the voltage by 1000 times.

The contact force at the end of a soft gripper is a key parameter for measuring its grasping ability. The AT8301 sensor measures the end contact force. Its maximum value is 1 N, and the measurement accuracy is 1 mN.

### 4.3. Analysis of bending displacement results for a single soft gripper

To verify the mechanical simulation performance of the soft gripper on different honeycomb pre-compression displacement and pre-stretching ratios of dielectric elastomer, the bending displacements of a single gripper finger are analyzed. The experiments are repeated twice to eliminate the influence of random errors and variations in manufacturing processes, thereby enhancing the reliability of the data.

#### 4.3.1. Experimental results analysis of a single soft gripper on different structural parameters.

**Fig 21:**
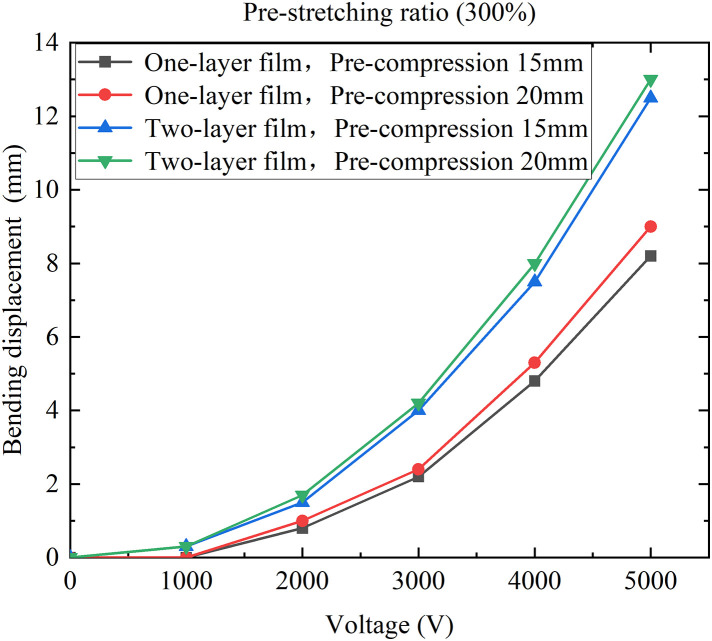
Experimental results of the honeycomb structure on different compressions.

Based on the simulation analysis of different pre-compression displacement of the honeycomb structure in Section 3.4.2, the film’s pre-stretching ratio is fixed at 300%. Experiments are then conducted to test the soft gripper under four sets of conditions: honeycomb pre-compression displacement of 15 mm and 20 mm, combined with one-layer and two-layer films respectively. The experimental results are illustrated in Fig 21.

In Fig 21, the blue and green lines represent experimental results with pre-compression displacement (15 mm and 20 mm) of the honeycomb structure with two-layer films, while the black and red lines show corresponding results for one-layer films, respectively. The figure demonstrates that soft grippers made with two-layer films exhibit greater bending displacements than those with one-layer films, regardless of pre-compression displacements. Under identical film layer counts, soft grippers with 20 mm show larger bending displacements compared to those with 15 mm pre-compression. When the two-layer film is stretched to 300% and the pre-compression displacement is 20 mm, the soft gripper achieves maximum bending displacement of 13 mm.

**Fig 22:**
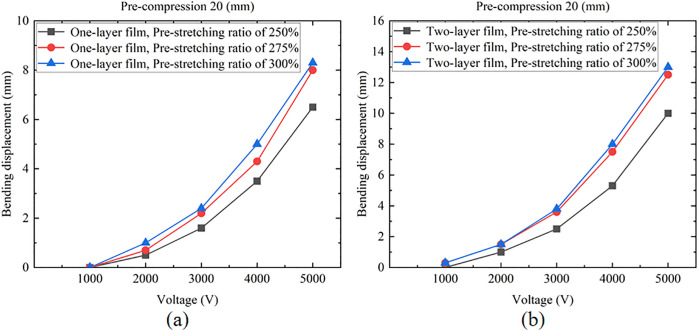
Experimental results at different pre-stretching ratio. (a) one-layer film. (b) two-layer films.

According to the simulation analysis of the film under different pre-stretching ratio in section 3.4.3, with the pre-compression displacement of the honeycomb structure fixed at 20 mm, soft grippers are tested on different pre-stretching ratio and different layers of the films. The experimental results are shown in Fig 22.

In Fig 22, the blue, red, and black lines represent the displacements at the pre-stretching ratios of 250%,275% and 300% for the films, respectively. Fig 22(a) shows the results for a single gripper made with a one-layer film, and the results for a single gripper constructed with two-layer film in Fig 22(b). The figure reveals that as the pre-stretching ratio increases, the bending displacement of the soft gripper progressively grows. Notably, bending displacement of the gripper with the two-layer configuration is significantly larger compared to that with the one-layer version. Consequently, when the pre-stretching ratio reaches 300% with two-layer film structures, the soft gripper achieves its maximum bending displacement of 13 mm.

#### 4.3.2. Comparison of data results and test performance of the soft gripper.

**Fig 23:**
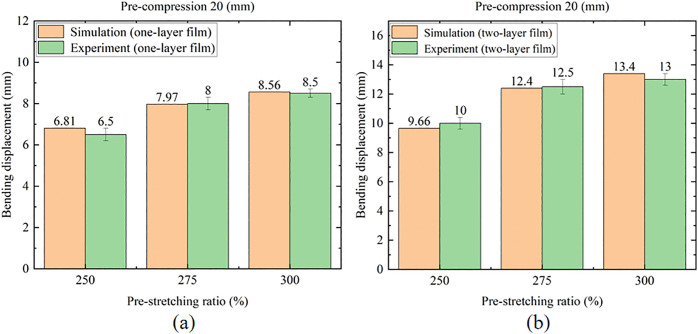
Comparison of simulation and experimental results. (a) one-layer film. (b) two-layer films.

To verify the accuracy of the improved design for soft gripper, a comparison between the simulation and experimental data of the gripper is conducted based on the aforementioned analysis. With the pre-compression displacement of the honeycomb structure fixed at 20 mm, the comparison results are illustrated in Fig 23.

As observed in Fig 23, when the pre-stretching ratio of the two-layer film is 300%, the maximum error between the simulation and experimental data is 0.4 mm, corresponding to 3.1% of the experimental result. The close agreement between the simulation and experimental data confirms the rationality of the proposed improvement of the soft gripper.

To systematically investigate the repeatability of the soft gripper, we select a specific set of structural parameters for validation. Specifically, a soft gripper is fabricated using one-layer dielectric elastomer film with a pre-stretching ratio of 275% and a honeycomb structure with a pre-compression of 20 mm. Subsequently, cyclic actuation tests are conducted to examine its bending displacement under repetitive electrical stimulation.

**Fig 24:**
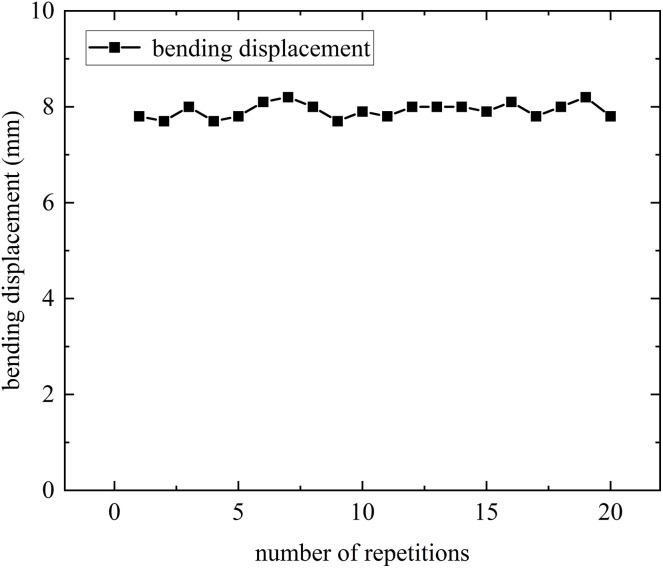
Variation of bending displacement with the number of cycles for the soft gripper.

In the experiment, each soft gripper is subjected to high-voltage excitation that increases at a constant rate. A total of 20 complete electrical loading and unloading cycles are performed, and the maximum bending displacement for each cycle is recorded, as shown in Fig 24.

The experimental results indicate that over the 20 consecutive tests, the bending displacement of the soft gripper remains consistently around 8 mm, showing no significant attenuation or drift. This demonstrates good data repeatability. These findings verify the stability and reliability of the proposed soft gripper under repetitive actuation, thereby confirming the credibility of the experimental data.

#### 4.3.3. Experimental results analysis of the bending deformation and contact force of a soft gripper.

**Fig 25:**
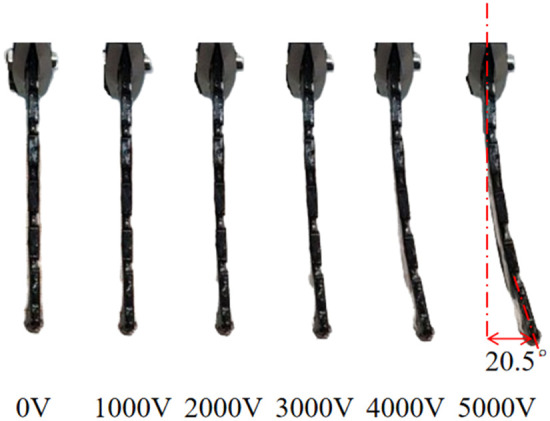
Bending deformation of soft gripper.

According to the above improved parameters for pre-stretching ratio 300% of the two-layer film and pre-compression displacement 20 mm of the honeycomb structure, the soft gripper is tested with the bending deformation in different voltages illustrated in Fig 25.

As illustrated in Fig 25, the bending displacement of the soft gripper increases gradually with the applied voltage, reaching a maximum of 13 mm at 5000 V. Specifically, the displacement increases moderately between 0 V and 3000 V, but it rises more rapidly in the range of 3000 V to 5000 V. As shown in the same figure, the maximum bending angle is 20.5° for the soft gripper whose length is 60 mm. Additionally, due to the electroactive deformation characteristics of the film, the honeycomb structure elongates slightly as the film deforms, resulting in a certain degree of elongation of the soft gripper.

**Fig 26:**
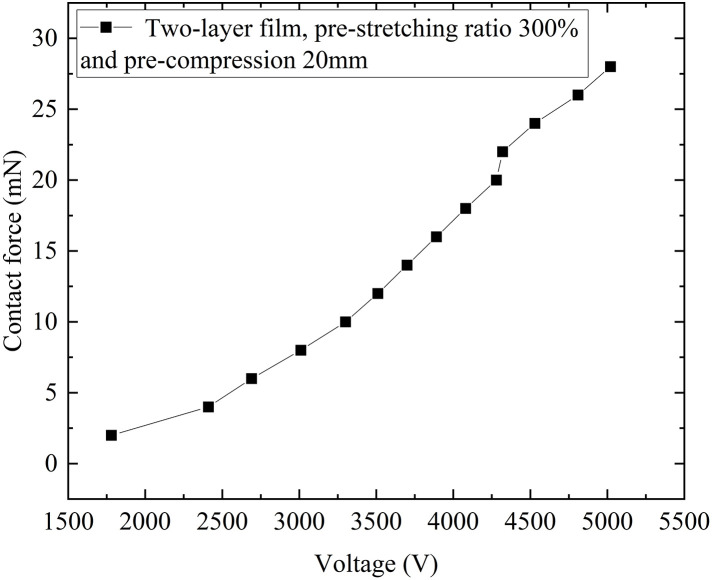
Contact force results of the soft gripper.

The contact forces of the above soft gripper are measured and the results are shown in Fig 26. When the voltage of the soft gripper is below 1500 V, the contact force at the end is 0 mN. When the voltage is below 3500 V, the contact increases slowly. It is observed that the change of the contact force is approximately linear in the voltage range of 2500 V and 5000 V. The maximum contact force is 28 mN at 5000 V.

### 4.4. Grasping experiment of soft gripper

To verify the grasping performance of the improved soft gripper, performance tests are conducted on various target objects. First, two identical single gripper fingers with excellent grasping capabilities are fabricated. Based on the bending deformation test results of the single gripper finger, the improved manufacturing parameters are determined as follows: a 300% pre-stretching ratio for the two-layer film and a 20 mm pre-compression displacement for the honeycomb structure. Next, the fabricated components are assembled into the complete soft gripper in accordance with the structural diagram (Fig 13), with the non-electrode-coated side facing the target object. Finally, the distance between the two gripper fingers is adjusted according to the size of the target object, enabling the soft gripper to achieve stable grasping.

**Fig 27:**
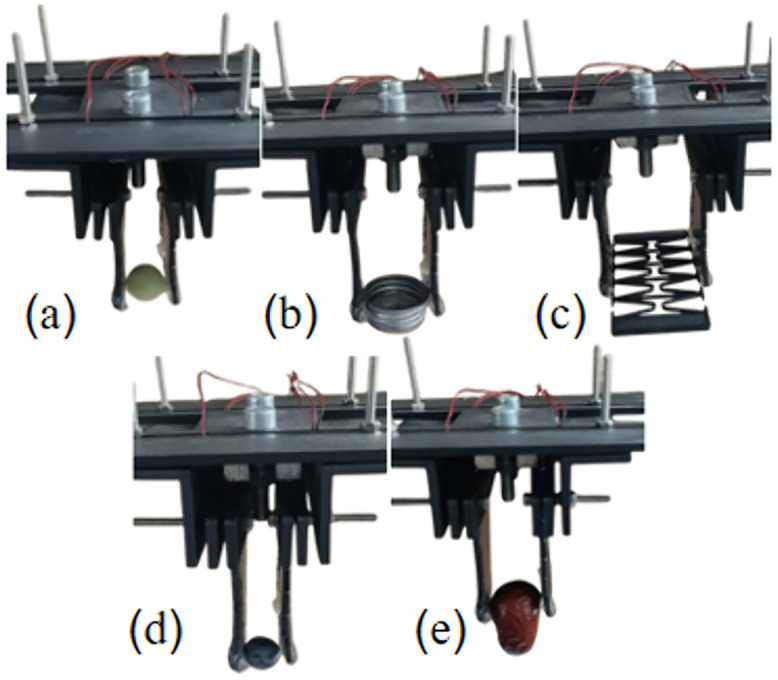
Schematic diagram of object capture.

The weight of a single gripper designed in the experiment is 4.6 g. The soft gripper is used to grasp objects of different textures and shapes, and the results are shown in Fig 27. Fig 27(a) displays a green elastic ball weighing 3.1 g; (b) a gray bottle cap weighing 2.7 g; (c) a honeycomb-supported structure weighing 3 g; (d) a blueberry weighing 1.2 g; and (e) a date weighing 5 g. Experimental results demonstrate that the proposed soft gripper, based on a dielectric elastomer actuator with a quadrangular star honeycomb support structure, is capable of grasping objects with various materials and shapes.

## 5. Conclusion

The paper presents a soft gripper driven by dielectric elastomers and supported by a honeycomb structure. A quadrangular star-shaped honeycomb structure with zero Poisson’s ratio was designed, followed by mechanical analysis and simulation. Based on the deformation and bending characteristics of the film and the soft gripper, the honeycomb structure was improved to achieve effective integration with the film, and the entire soft gripper was further improved. Considering the effects with the pre-stretching ratio of the film and the pre-compression displacement of the honeycomb structure on the bending performance of the single gripper, improved parameters were determined through experiments to enhance its bending performance. Finally, a complete soft gripper was fabricated. Experimental results demonstrated that the gripper successfully grasped various objects, with a maximum load-bearing capacity of 5 g. The proposed structural for dielectric elastomer-driven soft grippers supported by quadrangular star-shaped honeycombs provides a novel approach for soft gripper research.
